# Influencing factors of hospitalization costs for intensive rehabilitation in patients with post-stroke disorder of consciousness

**DOI:** 10.3389/fpubh.2025.1552148

**Published:** 2025-03-03

**Authors:** Miao Yu, Zhongmou Huang, Yansui Yang, Yulin Wang, Hai Ren, Shilan Tang

**Affiliations:** ^1^School of Healthcare Management, Tsinghua University, Beijing, China; ^2^School of Public Policy and Management, Tsinghua University, Beijing, China; ^3^Institute for Hospital Management, Tsinghua University, Shenzhen, China; ^4^Administrative Office, Shenzhen Longcheng Hospital, Shenzhen, China; ^5^General Management Department, Shenzhen Dapeng New District Medical and Health Group, Shenzhen, China

**Keywords:** disorders of consciousness, stroke, intensive rehabilitation, hospitalization costs, length of stay

## Abstract

**Objective:**

In China, patients requiring intensive rehabilitation often face a gap between acute treatment and sub-acute rehabilitation. This study evaluates the composition and determinants of post-acute hospitalization costs in stroke patients with disorders of consciousness (DoC).

**Methods:**

Data from 133 stroke patients with DoC who underwent inpatient rehabilitation at a tertiary hospital from 2015 to 2020 were collected, including demographic characteristics, clinical features, and hospitalization costs. Descriptive statistical analysis and univariate analysis were performed, followed by path analysis and Bootstrap mediation tests to explore factors influencing hospitalization costs.

**Results:**

The median hospitalization costs were $56,860.80. Rehabilitation costs accounted for the largest proportion of total hospitalization costs (36.55%). Direct factors influencing total costs included payment method, admission to the intensive care unit (ICU), pulmonary infection, and length of stay (LOS) (*p* < 0.05). The effect sizes ranked in descending order were LOS, ICU experience, payment method, and pulmonary infection. Bootstrap mediation tests revealed significant mediation effects (*p* < 0.05) of payment method, occupation, patient origin, hypertension, ICU experience, and death on total costs, indicating that these factors indirectly influenced costs by affecting LOS.

**Conclusion:**

Greater attention should be given to meeting the rehabilitation needs of patients by expanding resources for intensive rehabilitation and ensuring continuous rehabilitation services. Comprehensive and effective measures should be implemented to address cost-influencing factors early, without compromising the quality of care.

## 1 Introduction

Intensive rehabilitation refers to specialized therapeutic interventions designed for critically ill patients, facilitating their transition from the intensive care unit (ICU) to general wards and preparing them for long-term care (1, 2). This approach stabilizes patient conditions, reduces ICU length of stay, alleviates the burden on general wards, and optimizes the allocation of healthcare resources (3). Stroke is the second leading cause of death and the third leading cause of disability in the world (4). Rehabilitation remains an essential and indispensable component of care for stroke patients (5). Professional rehabilitation has been demonstrated to effectively improve functional outcomes in stroke patients (6), yet it also represents a significant portion of healthcare costs, particularly post-acute care costs excluding the acute treatment phase (7). In Europe, annual expenditures for stroke management are estimated at approximately €60 billion, with the average total cost per patient at €13,138.97. Treatment and rehabilitation make up the largest share, reaching 45 and 33%, respectively, in the first year (8, 9). In the United States, the average first-year medical cost for a stroke patient is $70,601, with rehabilitation expenses contributing the largest proportion (66%) (10). Studies suggest that approximately 10% of stroke patients suffer from disorders of consciousness (DoC), incurring a medical cost more than 10 times that of typical stroke cases (11–13).

Compared to other stroke patients, the condition of DoC patients is more complex and can typically be categorized into three levels based on the degree of consciousness loss: coma, persistent vegetative state (PVS), and minimally conscious state (MCS), with a higher degree of uncertainty in prognosis. During the post-acute rehabilitation phase, awakening these patients presents significant challenges, as they remain in a prolonged comatose state with low functional status, no ability for independent living, and are bedridden year-round. Additionally, 98% of these patients suffer from complications such as pulmonary infections (14, 15). Given these complexities and challenges, patients with DoC exhibit unique characteristics in clinical treatment, necessitating long-term rehabilitation interventions and significant investment of medical resources. Rehabilitation resources are scarce (5), and access to rehabilitation services varies significantly across healthcare systems, particularly in low- and middle-income countries, where high costs and continuity challenges further complicate the rehabilitation process (16). In China, the number of stroke survivors exceeds 28 million, accounting for approximately 28% of the global total of 101 million cases, making it the highest in the world (17). Annual rehabilitation expenditures for stroke patients in China amount to billions of dollars. Despite this, China has yet to establish a comprehensive three-tiered rehabilitation medical service system. The absence of intensive rehabilitation services has resulted in “prolonged hospitalization” for critically ill patients, such as stroke patients with DoC, during their transition from ICU acute to sub-acute care. This phenomenon imposes a significant economic burden. With the deepening of population aging, healthcare expenditures for such critically ill patients are expected to rise substantially in the future (18), posing immense financial challenges for both patients and health insurance funds.

Currently, China’s healthcare reform policies, which aim to reduce costs and improve services, have paid limited attention to high-cost patient populations like those with severe stroke. Focusing on such specific high-cost groups may hold greater potential for reducing medical expenses and enhancing the rehabilitation healthcare system (19). Despite the relatively low prevalence of DoC cases, the medical costs associated with these patients are disproportionately high. This highlights the necessity for in-depth research into the composition of hospitalization costs and the factors influencing them. Such research is crucial for optimizing the allocation of medical resources and enhancing the quality of rehabilitation services.

## 2 Materials and methods

### 2.1 Data

This study included stroke patients with DoC who underwent inpatient rehabilitation at a tertiary rehabilitation hospital between 2015 and 2020. Key information, including gender, age, occupation, origin, payment method, clinical diagnosis, device dependence, length of stay (LOS), and hospitalization costs, was extracted from the hospital’s electronic medical record system. The inclusion criteria were as follows: (1) primary diagnosis of stroke; (2) Loss of consciousness was diagnosed as coma, PVS, or MCS; and (3) age 18 years or older. Additionally, patients with incomplete data were excluded. Based on these criteria, a total of 133 patients were included in the study.

### 2.2 Variable selection

Previous research has shown that the primary factors influencing healthcare resource utilization include LOS, age, type and severity of disease, functional status at admission, payment method, and the presence of comorbidities or complications (20–23). In this study, hospitalization costs were set as the dependent variable. Costs were converted to U.S. dollars (US$) at an average exchange rate of US$1 = 6.6727 (from 2015 to 2020). Independent variables included gender, age, marital status, patients’ place of residence, occupation type, payment method, and the presence of comorbidities or complications. Comorbidities and complications analyzed included hypertension, diabetes, cardiovascular diseases, other diseases, pulmonary infections, epilepsy, electrolyte metabolism disorder, urinary tract infections, and other conditions. Device dependence factors considered were ICU experience, nasogastric tube dependence, ventilator dependence, tracheostomy tube dependence, and hyperbaric oxygen chamber dependence. Additionally, the study considered whether the patient regained consciousness or died during hospitalization.

### 2.3 Statistical methods

The data were analyzed using descriptive analysis and one-way analysis of variance. Variables with significant results in the one-way analysis of variance were further screened, with number of LOS as a mediating variable. To assess the mediation effects, we conducted path analysis and a bootstrap mediation effect test. As the total hospitalization cost and the number of LOS showed skewed distributions, raw data were log-transformed before analysis.

## 3 Results

### 3.1 Basic information

This study analyzed data from 133 patients, with males comprising 72.18%, significantly outnumbering females (27.82%). The majority of patients were older adult, with a mean age of 60.89 years (±15.12). The predominant payment method was medical insurance, utilized by 90.98% of patients. Occupational were primarily urban employees (33.08%) and retirees (36.84%). Most patients were local residents (52.79%). Hemorrhagic stroke was the most common type, representing 74.44% of cases, reflecting more severe brain injuries and a higher disease burden in these patients. Hypertension emerged as the most prevalent comorbidity, affecting 67.67% of patients, indicating a high level of comorbid complexity. Among complications, pulmonary infections were the most frequent, occurring in 38.35% of cases. Furthermore, 60.15% of patients experienced ICU transfers during hospitalization and demonstrated varying degrees of device dependence, highlighting the severity of their clinical conditions.

Nonparametric tests, including Mann–Whitney U and Kruskal-Wallis H tests, were employed to examine differences in LOS, total hospitalization costs, and rehabilitation costs (the largest cost component) across patient subgroups. The results (Table 1) revealed significant variations in LOS among patients with different payment methods, occupations, origins, hypertension status, ICU experience, and mortality status (*p* < 0.05). Similarly, total hospitalization costs varied significantly across subgroups categorized by payment method, occupation, origin, hypertension, pulmonary infections, ICU experience, and mortality (*p* < 0.05). Rehabilitation costs also exhibited significant differences based on payment method, occupation, origin, and hypertension status (*p* < 0.05).

**Table 1:** Sample descriptive statistics and results of one-way analysis of variance (*n* = 133).

Variable		Frequency	LOS	Hospitalization costs	Rehabilitation costs
Gender	Female	37 (27.82%)	−0.39	0.58	0.57
	Male	96 (72.18%)			
Age	≤35	6 (4.51%)	5.77	7.11	7.09
	36 ~ 50	26 (19.55%)			
	51–65	49 (36.84%)			
	66–80	34 (25.56%)			
	≥81	18 (13.53%)			
Payment method	Out of pocket	12 (9.02%)	3.24***	3.67***	3.91***
	Medical insurance	121 (90.98%)			
Marital status	Wedding	115 (86.47%)	0.93	0.59	0.54
	Others	18 (13.53%)			
Occupation	Urban workers	44 (33.08%)	15.73***	17.26***	9.37**
	Retirees	49 (36.84%)			
	a private firm (PRC usage)	19 (14.29%)			
	Unemployed and others	21 (15.79%)			
Origin	Local	70 (52.79%)	3.36***	3.98***	2.32**
	Non-local	63 (47.21%)			
Type of diagnosis	Ischemic stroke	34 (25.56%)	−1.31	−1.33	−0.62
	Hemorrhagic stroke	99 (74.44%)			
Number of comorbidities and complications	≤3	46 (34.59%)	1.58	1.92	0.51
	4 ~ 7	39 (29.32%)			
	≥8	48 (36.09%)			
Diabetes	No	99 (74.44%)	1.35	1.05	0.77
	Yes	34 (25.56%)			
Hypertension	No	43 (32.34%)	2.87***	2.70***	2.28**
	Yes	90 (67.67%)			
Cardiovascular disease	No	104 (78.20%)	1.08	1.26	0.26
	Yes	29 (21.80%)			
Any other co-morbidities	No	81 (60.90%)	−1.69*	−1.36	−1.93
	Yes	52 (39.10%)			
Pulmonary infection	No	82 (61.65%)	1.68*	2.33**	1.21
	Yes	51 (38.35%)			
Epileptic	No	107 (80.45%)	0.41	0.88	0.53
	Yes	26 (19.55%)			
Electrolyte metabolism disorder (EMSD)	No	112 (84.21%)	−0.08	−0.42	−0.11
	Yes	21 (15.79%)			
Venous thrombosis	No	126 (94.74%)	−0.33	−0.09	−0.37
	Yes	7 (5.26%)			
Urinary tract infection	No	122 (91.73%)	−0.76	−0.64	−0.96
	Yes	11 (8.27%)			
Any other complications	No	124 (93.23%)	−0.76	−0.51	−0.27
	Yes	9 (6.77%)			
ICU experience	No	80 (60.15%)	2.26**	4.27***	1.95*
	Yes	53 (39.85%)			
Nasogastric catheter dependent	No	25 (18.80%)	−0.06	0.09	−0.94
	Yes	108 (81.20%)			
Ventilator dependent	No	109 (81.95%)	−1.36	−0.58	−1.79*
	Yes	24 (18.05%)			
Tracheal catheter dependent	No	40 (30.08%)	−0.60	−0.27	−0.88
	Yes	93 (69.92%)			
Hyperbaric chambers dependent	No	86 (30.08%)	−1.35	−1.50	−1.05
	Yes	47 (35.34%)			
Awake	No	115 (86.50%)	−0.21	−0.42	0.22
	Yes	18 (13.50%)			
Death	No	120 (90.23)	2.25**	2.19**	1.09
	Yes	13 (9.77%)			

*p < 0.1, **p < 0.05, ***p < 0.01. ICU, intensive care unit; PRC, People’s Republic of China; LOS, length of stay; EMSD, electrolyte metabolism disorder.

### 3.2 The components of hospitalization costs

The average LOS was 400.71 days. The average hospitalization cost was $86,048.75, with a median cost of $56,860.80. Among these, the average rehabilitation cost was $31,453.35. In the composition of hospitalization costs, rehabilitation, drug, and treatment expenses accounted for the top three proportions of total costs, at 36.55, 17.11, and 12.86%, respectively (Table 2).

**Table 2:** Components of hospitalization costs.

Variables		Average value	Standard deviation	Median	Composition ratio (%)
Hospitalization costs		86,048.75	85,926.42	56,860.80	100.00
	Diagnostic costs	983.64	1,131.15	660.00	1.14
	Drug costs	14,714.31	19,626.69	7,974.70	17.11
	Inspection and testing costs	8,553.57	11,643.75	4,673.85	9.94
	Treatment costs	11,065.96	15,337.53	6,195.44	12.86
	Injection costs	508.26	716.00	250.96	0.59
	Rehabilitation costs	31,453.35	37,127.15	18,648.75	36.55
	Bed charge	5,048.91	6,501.09	3,089.00	5.87
	Nursing costs	8,508.81	9,072.01	5,500.77	9.89
	Surgical anesthesia costs	81.53	182.05	14.99	0.09
	Blood transfusion costs	59.46	238.01	0.00	0.07
	Material costs	4,137.85	4,601.12	2,468.37	4.81
	Other costs	933.09	1,568.59	307.22	1.08

From different cost percentiles, patients in the top 50% of hospitalization costs accounted for 83.98% of the total hospitalization costs. Patients in the top 25, 10, 5, and 1% of hospitalization costs contributed to 58.99, 32.26, 18.10, and 4.56% of the total expenses, respectively. This indicates that a small number of high-cost patients consumed the majority of healthcare resources (Table 3).

**Table 3:** Percentage of total hospitalization costs for patients in different percentiles.

Percentile	Hospital cost	Sample size	Percentage of hospital costs above the percentile
50%	56,860.80	66	83.98%
75%	118,772.12	33	58.99%
90%	214,376.12	13	32.26%
95%	261,402.40	4	18.10%
99%	490,823.30	1	4.68%

### 3.3 Path analysis

**Figure 1:**
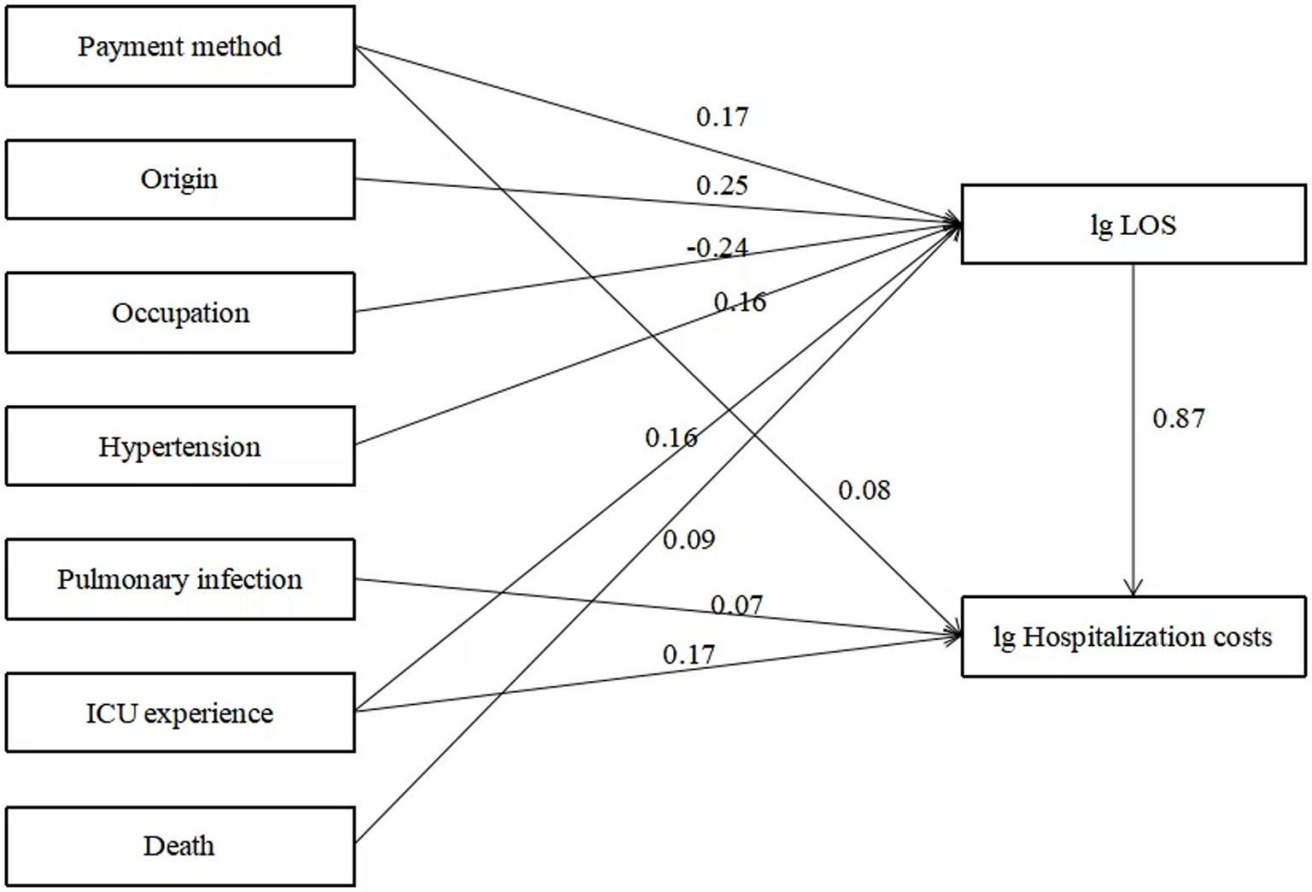
Path analysis of factors influencing hospitalization cost. ICU, intensive care unit; LOS, length of stay.

Using hospitalization costs as the dependent variable and LOS as the mediating variable, a path analysis model was constructed by including variables with statistical significance (*p* < 0.05) identified in the univariate analysis. After multiple adjustments, the standardized coefficients for each path are presented in Figure 1. According to the path analysis results (Table 4), payment method, ICU transfers, pulmonary infections, and LOS directly influenced total hospitalization costs (*p* < 0.05), with the effect sizes ranked in descending order as LOS, ICU experience, payment method, and pulmonary infections.

**Table 4:** Path analysis of factors influencing hospitalization costs.

Path	b’	S.E.	C.R.	*p*
Payment method → lg LOS	0.17	0.12	2.27	0.02
Origin→ lg LOS	0.25	0.07	3.29	<0.001
Occupation → lg LOS	−0.24	0.03	−3.14	<0.001
Hypertension → lg LOS	0.16	0.08	2.04	0.04
Pulmonary infection → lg LOS	0.13	0.07	1.67	0.10
ICU experience → lg LOS	0.16	0.07	2.05	0.04
Death → lg LOS	0.09	0.1	1.22	0.03
Payment methods → lg Hospitalization costs	0.08	0.05	2.59	0.01
Origin→ lg Hospitalization costs	0.05	0.03	1.74	0.08
Occupation → lg Hospitalization costs	0.02	0.01	0.66	0.51
Hypertension → lg Hospitalization costs	−0.02	0.03	−0.77	0.44
Pulmonary infection → lg Hospitalization costs	0.07	0.03	2.39	0.02
ICU experience → lg Hospitalization costs	0.17	0.03	5.77	<0.001
Death → lg Hospitalization costs	−0.01	0.04	−0.4	0.69
lg LOS → lg Hospitalization costs	0.87	0.03	26.25	<0.001

CR, critical ratio; ICU, intensive care unit; SE, standard error; LOS, length of stay.

### 3.4 Bootstrap mediated effects test

We investigated whether the independent variables, in addition to their direct impact on total hospitalization costs, also exerted an indirect influence through LOS, thus demonstrating a mediating effect. Using the bootstrap test for mediation effects in Amos 24.0, we found that payment method, occupation, origin, hypertension, ICU experience, and mortality significantly mediated hospitalization costs by indirectly influencing the number of LOS. The total effects analysis revealed that the variables influencing hospitalization costs, ranked in descending order, were LOS, ICU experience, payment method, origin, hypertension, mortality, and occupation (Table 5).

**Table 5:** Results of bootstrap mediation effect test for lg LOS.

Direct effect			Indirect effect		95% CI		Aggregate effect	
Path	Effect size	*p*	Path	Effect size	Boot CL lower limit	Boot CL upper limit	Effect size	*p*
Payment methods →lg Hospitalization costs	0.08	0.03	Payment method →lg LOS → lg Hospitalization costs	0.25	0.34	1.32	0.32	<0.001
–	–	–	Occupation →lg LOS → lg Hospitalization costs	−0.18	−0.34	−0.09	−0.18	<0.001
–	–	–	Origin →lg LOS → lg Hospitalization costs	0.27	0.25	0.81	0.27	0.02
–	–	–	Hypertension →lg LOS → lg Hospitalization costs	0.23	0.15	0.82	0.23	0.01
Pulmonary infection →lg Hospitalization costs	0.07	0.03	Pulmonary infection →lg LOS → lg Hospitalization costs	0.09	−0.14	0.51	0.16	0.04
ICU experience→lg Hospitalization costs	0.17	<0.001	ICU experience→lg LOS → lg Hospitalization costs	0.20	0.14	0.63	0.37	<0.001

ICU, intensive care unit; LOS, length of stay.

## 4 Discussion

### 4.1 Direct effects

LOS is the most significant and direct factor influencing hospitalization costs (0.87), serving as both a primary contributor and a mediating variable for the effects of other factors. LOS reflects a hospital’s internal management efficiency and offers an effective means for reducing hospitalization costs. It is not only directly tied to a patient’s financial burden but also contributes to potential resource wastage (21). In this study, the average LOS reached an exceptionally high 400.71 days, likely due to gaps in the rehabilitation healthcare continuum. This phenomenon, known as “retention,” arises from poor communication between departments, information transfer failures, or inefficient resource allocation, which hinder the smooth transition of patients from one treatment stage to another, ultimately impacting therapeutic outcomes and recovery progress. ICU directly influence hospitalization costs (0.17) and indirectly affect costs through LOS (0.20). Studies indicate that ICU care improves survival rates and functional outcomes but comes with significant financial implications (24). Extended ICU stays can lead to involuntary overutilization of medical care, resulting in severe resource wastage. This issue is closely linked to the structure of the rehabilitation healthcare system, hospital management, physician practices, and family decision-making authority (13). Our findings underscore the importance of controlling unnecessary ICU retention and establishing a scientifically sound “bridge” for patient transitions between ICU and general wards (25, 26). Involuntary overutilization differs from proactive overutilization driven by factors such as physician incentives; it stems from systemic shortcomings, such as gaps in service capabilities and content. This not only exacerbates patients’ financial burdens but also leads to resource inefficiency (27). These findings highlight the urgent need to integrate acute and subacute rehabilitation services, reducing healthcare system strain while alleviating patients’ economic burden (28).

Payment method directly affects hospitalization costs (0.08) and indirectly influences costs through LOS (0.25). Patients covered by insurance generally incur higher hospitalization costs and longer LOS compared to self-paying patients (11). China has constructed the world’s most extensive basic medical security network, with the coverage rate of basic medical insurance exceeding 95% and the number of insured individuals surpassing 1.334 billion. This has essentially accomplished the goal of universal health insurance coverage. Nevertheless, despite the high coverage rate, China’s medical insurance system still confronts several challenges. For instance, there are still certain populations not covered by medical insurance; there are considerable disparities in medical insurance policies between urban and rural areas as well as among different regions, resulting in imbalances in the level of protection offered. Payment mechanisms significantly impact hospitalization costs, with variations in financial incentives within the same system influencing hospitals’ service models. Adjusting payment structures can encourage resource allocation toward more cost-effective treatments (29). Research suggests that mixed payment models more effectively reflect actual needs and costs, optimizing the management of hospitalization costs (30).

Pulmonary infections directly affect hospitalization costs (0.07). Complications are among the most significant reversible factors in hospitalization costs. The greater the number of complications, the more uncertain the prognosis and the higher the required resource investment, ultimately driving up hospitalization costs (31). These findings emphasize the need for early prevention and management of complications in patient care (32).

### 4.2 Indirect effects

Patient origin indirectly influences hospitalization costs through its effect on LOS (0.27), with local patients incurring higher LOS and hospitalization costs. Research suggests that regional disparities are a major factor driving inequality in healthcare service utilization, with unequal distribution of healthcare resources contributing significantly to these differences (33). Additionally, non-local patients may have lower medical expenses and shorter LOS due to being transferred to local hospitals for further rehabilitation once their condition stabilizes. Other contributing factors include the financial burden of out-of-town treatment, a lack of suitable institutions for intensive rehabilitation, and uncertainties regarding prognosis, which may force families to forgo rehabilitation treatment. However, it is important to note that this study only accounted for the length of hospital stays and associated costs incurred by patients during their hospitalization at this institution from 2015 to 2020. It did not consider the time spent in rehabilitation or other facilities following transfer, which may lead to an underestimation of hospitalization costs for non-local patients.

Hypertension indirectly affects hospitalization costs through its impact on LOS (0.23), with hypertensive patients showing higher LOS and costs. Hypertensive patients often present with more complex conditions. Studies have indicated that hypertension and stroke severity are key factors driving high costs in stroke care (34). Older adults frequently suffer from multiple chronic comorbidities, leading to higher risks in treatment, rehabilitation, and medication. This adds complexity to the diagnostic, therapeutic, and rehabilitative processes (35). Comorbidities significantly increase hospitalization costs by prolonging LOS and increasing the use of medical resources. Future research could further investigate the specific impact of various types of comorbidities on hospitalization costs and propose targeted management strategies to mitigate these expenses.

Death indirectly influences hospitalization costs through LOS (0.17), with deceased patients incurring higher costs. Contrary to findings in other studies where mortality was associated with lower hospitalization costs, the observed discrepancy in this study may be attributed to prolonged hospital stays during the observation period. Surviving patients typically have a life expectancy of 2 to 5 years, and the likelihood of regaining consciousness decreases with the duration of coma (36). Consequently, these patients often require more extensive diagnostic tests, treatments, and complex rehabilitation interventions aimed at promoting recovery. Studies by Japanese scholar Yu Bingkuang (37) comparing short-term acute treatment costs with long-term care costs found that long-term care costs accounted for only 10–20% of short-term acute treatment expenses. The dramatic increase in individual short-term acute treatment costs typically occurs during end-of-life care and is largely unaffected by extended survival. As Bingkuang Yu’s research indicates, treatment costs often surge during critical, life-threatening moments, driven not by age or life expectancy but by end-of-life care needs. Influenced by traditional Chinese values like filial piety, families in China frequently pursue aggressive medical interventions for critically ill relatives, leading to significant increases in healthcare expenditures. Recent studies support this view, showing that mortality is often associated with complex treatment processes, prolonged LOS, and higher resource consumption. To reduce this economic burden, improvements in preventive measures, enhanced healthcare service efficiency, and comprehensive patient management are necessary (38, 39).

Occupation indirectly influences hospitalization costs through LOS (−0.18), with patients having lower economic security incurring lower hospitalization costs. Such patients may forgo treatment due to their inability to afford long-term, high-cost care. Patients in occupational categories such as urban employees and retirees, compared to those in other categories, tend to have stable income sources and stronger financial foundations to support their inpatient rehabilitation (12). In contrast, unemployed individuals or those without stable workplaces often face economic instability and greater risk factors, such as low health awareness, engagement in physical labor, and insufficient utilization of healthcare services. These factors, when accumulated over time, may lead to worsening conditions or late-stage disease discovery. These findings highlight the importance of strengthening primary healthcare services, paying attention to the distribution characteristics of different populations, and implementing targeted health management for key groups.

## 5 Conclusion

This study used stroke patients with DoC as an example to explore the composition and influencing factors of hospitalization costs through path analysis and Bootstrap mediation effect testing. The results showed that rehabilitation costs accounted for the largest proportion of total hospitalization costs. Payment method, ICU experience, pulmonary infections, and LOS had a direct impact on total hospitalization costs. Additionally, payment method, occupation, patient origin, hypertension, ICU experience, and death had significant mediation effects on total costs (*p* < 0.05), indirectly influencing hospitalization costs through LOS. Moving forward, it is crucial to address patients’ rehabilitation needs, expand resources for intensive rehabilitation care, and ensure the provision of continuous rehabilitation services. Comprehensive and effective measures should be implemented to intervene early in factors influencing hospitalization costs, without compromising the quality of care. These efforts will help curb the rapid increase in hospitalization costs and alleviate the economic burden on patients and society.

It should be noted that the trends in stroke incidence and prevalence among Chinese patients are generally similar to those observed in middle-income countries (18). Therefore, the findings of this study offer valuable insights for other developing countries with comparable socioeconomic conditions.

## Data Availability

The original contributions presented in the study are included in the article/supplementary material, further inquiries can be directed to the corresponding author.
